# Microstructural Characteristics of Arc Beads with Overcurrent Fault in the Fire Scene

**DOI:** 10.3390/ma13204521

**Published:** 2020-10-12

**Authors:** Zhijin Yu, Shuangshuang Chen, Jun Deng, Xueyan Xu, Weifeng Wang

**Affiliations:** 1 College of Safety Science and Engineering, Xi’an University of Science and Technology, Xi’an 710054, China; chenshuang19960819@163.com (S.C.); jnyangs171@163.com (J.D.); xyxu0108@163.com (X.X.); guyu199669@163.com (W.W.); 2 Mineral Engineering Postdoctoral Station, Xi’an University of Science and Technology, Xi’an 710054, China

**Keywords:** forensic science, arc beads, electrical fires, metallographic structure, elemental composition, overcurrent intensity

## Abstract

Arc beads are high-temperature luminous electric discharges that form across a gap between two bodies, which is one of the vital forensic pieces of evidence for the evaluation of electrical fires. In this study, based on an actual electrical fire, the microstructure of arc beads from a copper wire that experienced an overcurrent fault was investigated by optical microscopy and scanning electron microscopy. Moreover, the effects of the overcurrent intensity on the grain morphology, trace elements, and microstructure of the arc beads were analyzed. The results showed that the simulated metallographic structure of the arc beads is mainly dendrite at four times the rated current, which is consistent with the fire scene. With an increase in the overcurrent, the average diameter, perimeter, and area of the grains increased, while the dendrite growth was inhibited by the overcurrent. In addition, the main trace elements were Cu, C, O, and Cl. When the current increased, the Cu content gradually decreased and tended to be stable, while the C content gradually increased. The conclusion of this research provided a scientific reference for identifying the melting trace in a copper conductor under overcurrent fault.

## 1. Introduction

Electrical fire is one of the main causes of most urban building fires. From the statistics of the Emergency Management Department Fire and Rescue Bureau in China [1], in 2018 electrical fires accounted for 36.6% of the total number of fires in China. In these electrical fires, the main causes included short circuits, overload, poor contact, and electrical equipment failure, among which short circuits, neutral wire floats, and lightning strikes will all make an electrical system overcurrent fault [2,3,4,5]. Therefore, overcurrent fault is the final manifestation of a variety of electrical fire causes.

The temperature of copper wire will be elevated under overcurrent faults [6]. If continued for long periods of time, the conduction will be molten, and due to the released energy arc beads will be formed where the wires are melted. Analyzing the characteristics of arc beads at the fire scene will provide a reference for determining the cause of the fire [7,8,9,10]. Using optical microscopy (OM), Takak et al. [11] produced metallographic photographs of short-circuit arc beads for the first time and found that the short-circuit microstructure of an arc bead was composed of fine cellular crystals. Mo et al. [12] quantitatively analyzed the parameters of a metallographic structure in a short-circuit weld mark. Gao et al. [13] observed the short-circuit copper molten marks with OM and atomic force microscopy (AFM), verifying that AFM could be an auxiliary method to confirm the reasons for a fire. Lee et al. [14] and Liu et al. [15] investigated the metallographic structure and elemental composition of arc beads under a short-circuit with scanning electron microscope-energy dispersive spectroscopy (SEM-EDS). Using SEM, Gray [16] observed numerous square and rectangular pockmarks on fire-causing arc beads (FCABs). For distinguishing the primary and secondary arc beads, Howitt [17] and Erlandsson [18] analyzed the characteristics of fire marks to determine whether they are primary or secondary. Wu et al. [19,20] compared the distribution of the substances of Cu and Cu_2_O by x-ray photoelectron spectrometry (XPS). However, these studies focus on the study of trace characteristics in short-circuit electrical fires, which is fundamentally different from overcurrent faults, and thus the studies are not shared. He et al. [8] and Si et al. [21] investigated the molten thermoplastic drip and microstructure under an overcurrent fault. Gao et al. [22] investigated the heating process of the core under an overcurrent fault and the failure of insulation under thermal decomposition. However, the current results only focused on a small amount of experimental data and failed to simulate the field environment. Concrete scientific evidence still cannot be provided for cases related to overcurrent fault [23]. Furthermore, the effect of the overload current on the microstructural characteristics of arc beads was not analyzed.

In this paper, based on a machinery plant fire that occurred in Xi’an city, China, the characteristics of the traces of the fire scene were analyzed by OM and SEM-EDS, and the results were compared with experimental simulation traces to identify the cause of the fire. Subsequently, the effect of the overload current on the grain morphology, trace elements, and microstructure of the overcurrent arc beads were analyzed. Overall, our results provide clear scientific evidence to identify the melting trace in a copper conductor under overcurrent fault.

## 2. Fire Scene Observation

The examination of a machinery plant fire that occurred at 5 a.m. on 10 October 2018, in Xi’an, China, was completed by the local fire department. There was an ambient temperature of 283.5 K and a north wind of 1.5 m/s. The fire caused the severe burning of the plant and damaged the property; the loss was assessed to be ¥3,000,000, but there were no casualties. However, the information reported from the fire monitoring system and witness testimony did not pinpoint the origin of the fire.

**Figure 1:**
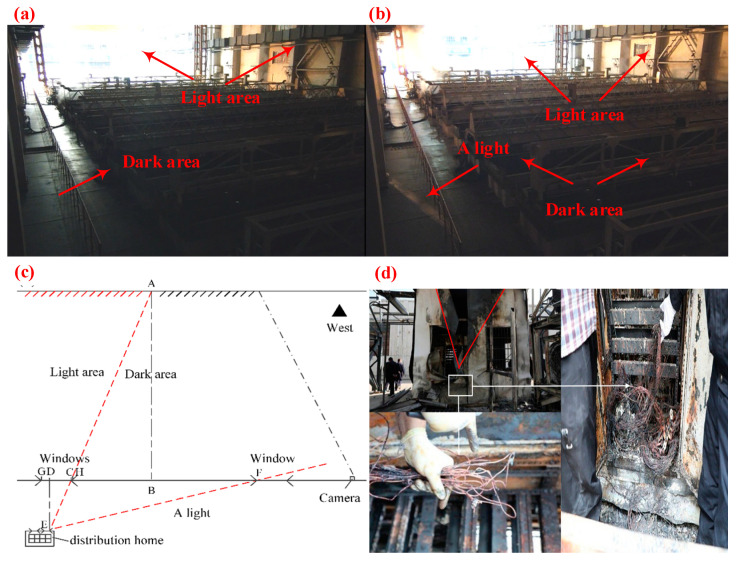
Images from the fire scene: (**a**,**b**) the frames of the video of fire scene at different times, (**c**) schematic of video images, and (**d**) melted traces that were found in the northwest corner of the distribution room.

The fire site monitoring was investigated, but the fire point was not directly found. Through video frame cutting technology, the Figure 1a shows that the transmitted light irradiated into the plant building at a certain moment, and a light area was formed. the distribution room located in the east was inferred to be a fire point. After 3 min and 58 s, Figure 1b shows a light suddenly appearing in the bottom left region of the video, and the light area became brighter. According to the principle of the linear propagation of light and the structure of the site, a video analysis diagram of the fire scene is drawn in Figure 1c. Line AEis associated with Line EF at point E, and based on the similarity principle of triangles (ΔABC ≈ ΔEDC); the Length of line CD is 3120 mm. When Line CD is an element of (2930 mm, 4160 mm), E is placed at the northwest window of the distribution room, and we doubt that E was the origin of the fire. A detailed inspection of the distribution room was carried out, as shown in Figure 1d. The west wall of the distribution room had a remarkable V-shaped crack that was peeling. It also can be seen in Figure 1d that the peeling crack mark combustion termination line differed with respect to a horizontal line. This is because the flames spread south under the influence of the wind from the north.

Subsequently, molten marks from the copper wire were found under the third distribution cabinet in the distribution room, as shown in Figure 1d. The molten marks that were extracted from the cabinet bottom and the macroscopic characteristics showed that the round, smooth shape of the molten marks is consistent with the characteristics of an overcurrent fault, as described in the National Fire Protection Association (NFPA) 921 [5]. However, the estimation was a subjective judgment of fire investigators, and scientific analyses of the artifacts and experimental verification are required.

## 3. Materials and Methods

**Figure 2:**
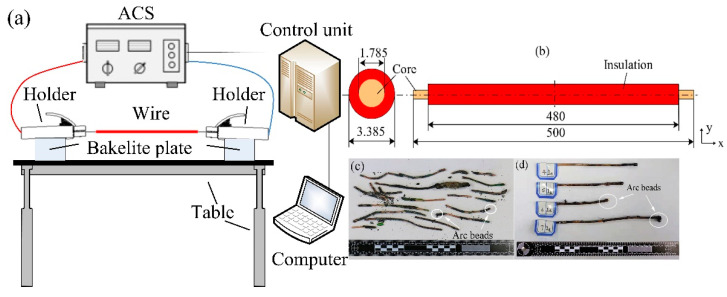
Images of the experimental system: (**a**) schematic of the experimental apparatus, (**b**) wire configuration (mm), (**c**) Overcurrent arc beads (OABs) from the fire scene, and (**d**) experimentally produced OABs.

To investigate the actual causes of the fire, Figure 2a indicates that the experimental apparatus consisted of an alternating current source (ACS), a control unit, a holder, sample wire, Bakelite plates, and a base. The ACS had a measurement range of 0–300 A with an accuracy of 0.1 A and supplied a steady current. The control unit was used to adjust the current in the wire. The sample wire was polyvinyl chloride (PVC) single copper wire, which was the same as the manufacturer and model in the fire scene. The wire parameters are shown in Table 1.

The holder was a quick-break structure for connecting the sample wire, and the Bakelite plates were used as an insulation material to prevent a ground fault. The base was made of stainless steel with a length of 1500 mm, width of 1000 mm, and height of 1200 mm.

Experiments have shown that, without excessive thermal insulation, it takes a 400–700% overcurrent to cause ignition [24]. The sample wire configuration is shown in Figure 2b. The ACS was used to simulate 400%, 500%, 600%, and 700% overcurrent faults. The fire scene was simulated by the experimental environment, and all the experiments were executed at 283.5 K and with a 65% relative humidity. Every experiment was repeated at least 10 times in an air-conditioned controlled room. In addition, the average means from 10 repetitions was used in the organized process of the experimental data to reduce error.

### OM/SEM-EDS Testing

The samples were prepared in accordance with the standard metallographic methods of the American Society for Testing and Materials (ASTM E3-11) [25] (standard practice for the preparation of metallographic specimens) and ASTM E407-07 [26] (standard practice for macroetching metals and alloys). Figure 2c,d show the overcurrent arc beads (OABs) sample. The arc beads were cut from the two ends on the gap side at the overcurrent site and then cast by plastic mounting (acrylic resin). The samples were ground with 240-, 800-, and 2000-grit sandpapers and polished with flannelette. The etching solution was a mixture of 100 mL of H_2_O, 50 mL of HCl, and 5 g of solid FeCl_3_, and the sample surface was immersed in this solution for a few seconds until the metallographic structure appeared; this process was repeated as necessary.

**Figure 3:**
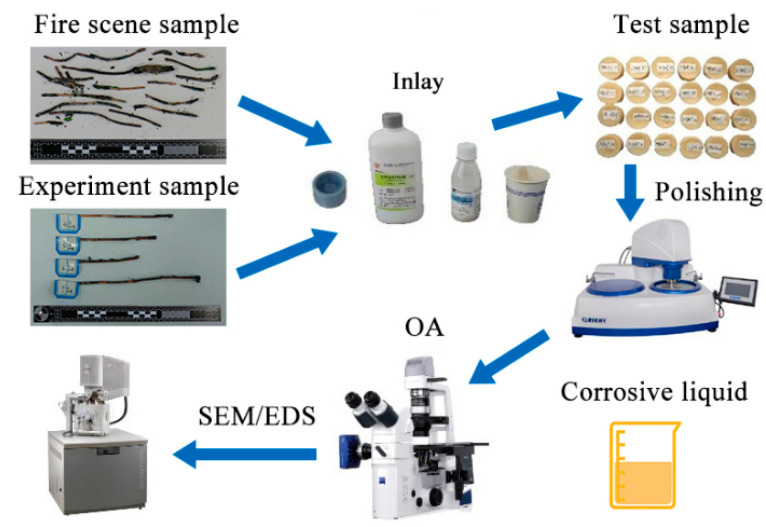
The preparation process of the arc beads from the fire scene and experiment.

Optical microscopy (Zeiss Inc. Axio Vert. A1. Shijiazhuang, China) and SEM (Thermo Fisher Scientific Inc. QUANTA FEG 450. Xi’an, China) were used to observe the microstructure of OABs, as shown in Figure 3. The Image Pro Plus software (Media Cybernetics, Rockville, MD, USA) was used to calculate the average diameters (D), perimeters (C), and areas (S) of the grains in the metallographic images. More than 200 grains were counted at each overcurrent (primary dendrites were removed).

## 4. Results and Discussion

### 4.1. Experimental Result

**Figure 4:**
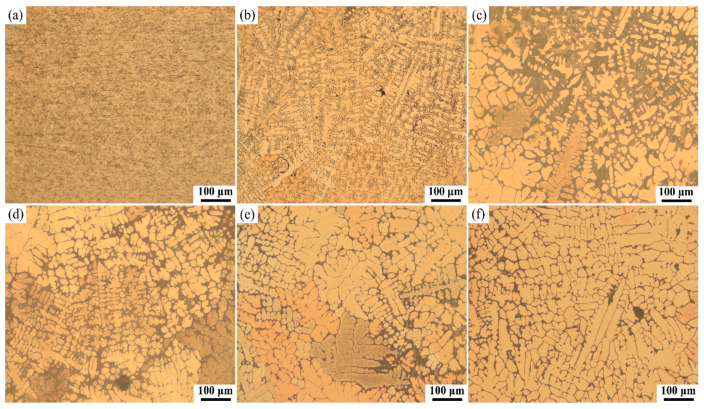
Cross-sectional metallographic images of copper wire under different conditions: (**a**) normal copper wire, (**b**) OABs from the fire scene, (**c**) after 400% overcurrent, (**d**) after 500% overcurrent, (**e**) after 600% overcurrent, and (**f**) after 700% overcurrent.

The metallographic characteristics of the copper wire under different conditions are shown in Figure 4. A metallographic image for a normal copper wire is shown in Figure 4a, and no obvious grains and grain boundaries were found. The metallographic image of the OABs from the fire scene is presented in Figure 4b, where it can be seen that the primary trunks of the dendrites were very directional, and there were many secondary dendrite branches. The experimental metallographic images from the experimental OABs (400% overcurrent) are shown in Figure 4c. The metallographic image shows dendrites that grew with a strong orientation, and its metallographic characteristics are basically consistent with those of the real fire site.

**Figure 5:**
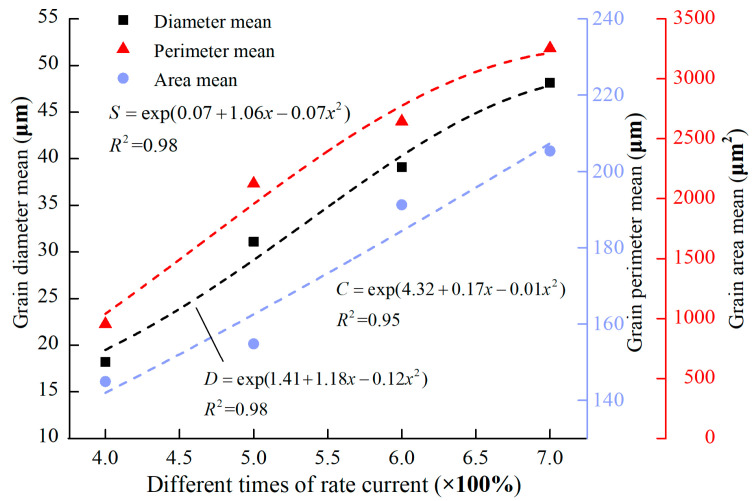
Grain characteristics of arc beads under different conditions of overcurrent.

Figure 5 shows the variation of the grain diameters (*D*), perimeters (*C*), and areas (*S*) under different conditions of overcurrent. The grain morphology changes with the increase in the current.

**Figure 6:**
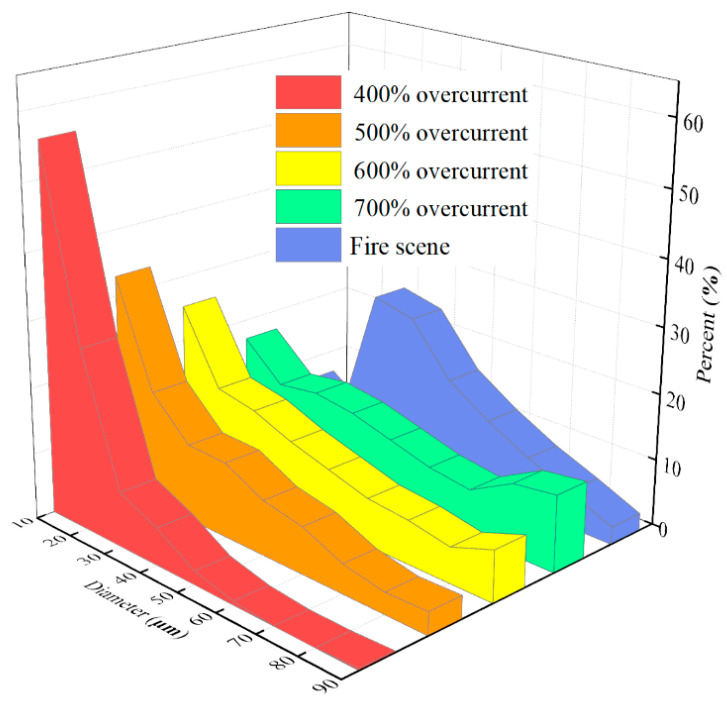
Variation in the average diameter of the OABs under various overcurrent intensities.

**Figure 7:**
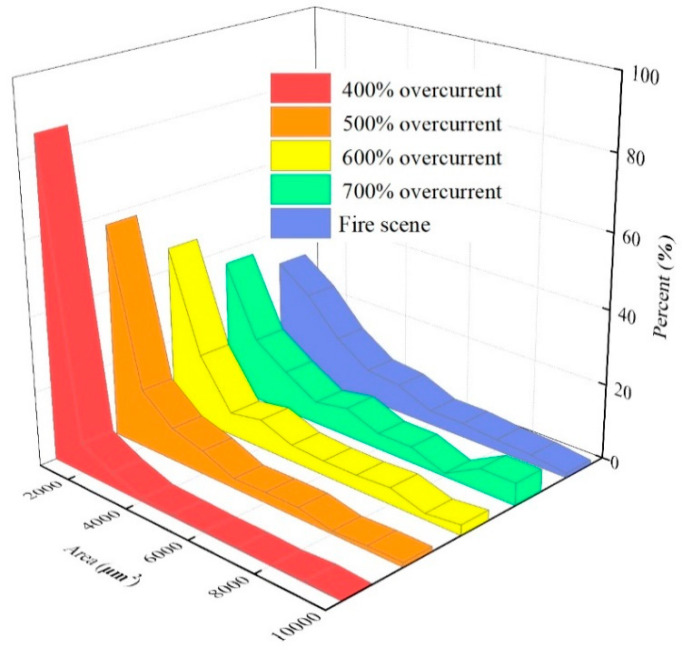
Variation in the average area of the OABs under various overcurrent intensities.

Figure 6 and Figure 7 show the variation in the specific average diameter and area of OABs versus the different overcurrent intensities. When the overcurrent was 400%, the grain percentage tended to reach zero as the diameter increased, which is similar to the average area. However, the distribution of diameter tends to be stable under the condition of high-power overcurrent, which means that the proportion of grains with a larger diameter and area increases, and with the increase in the overcurrent intensity the grain proportion of larger diameter and area increased, and with the increase in the overcurrent intensity the proportion of grains with a larger diameter and area also increased. Thus, the overcurrent intensity has a positive influence on the growth of the grain diameter and area. The average diameter of OABs at the fire scene was mainly 30 nm, because the OAB solidification was affected by external factors that interfered with the fire.

**Figure 8:**
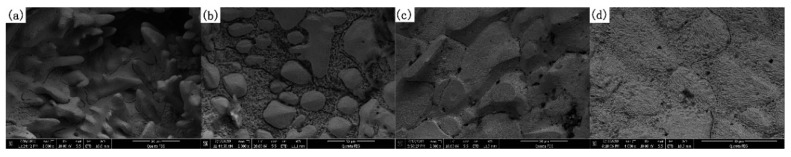
SEM analysis of OABs at different overcurrent intensities: (**a**) 400%, (**b**) 500%, (**c**) 600%, (**d**) 700%.

Figure 8 shows the microscopic morphology of the hole surface of the OABs versus different overcurrent intensities at a magnification of 2000×. At a 400% overcurrent, the inside of the holes was rough, the primary dendrites were sharp and developed, and the secondary dendrites were also developed.

**Figure 9:**
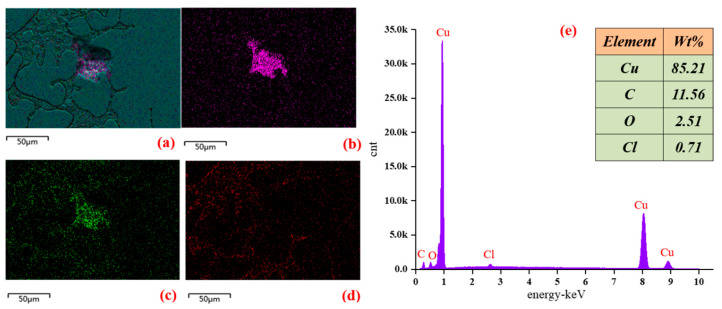
Energy dispersive spectroscopy spectrum for the 400% overcurrent of arc beads: (**a**) total element mapping, (**b**) content, (**c**) O mapping, and (**d**) C mapping. (**e**) element distribution mapping

Energy dispersive spectroscopy technology was used to obtain the contents of normal wires, fire traces, and simulated traces. The results of fire traces are shown in Figure 9.

The elements of the overcurrent fault trace are Cu, C, O, and Cl. The Cu and C contents are 85.21% and 11.56%, respectively. From Table 2, it can be found that the characteristics of the fire trace elements are similar to those of the 400% overcurrent trace elements.

### 4.2. Effect of the Current on the Metallographic Structure of OABs

The experimental metallographic images from the experimental OABs (400–700% overcurrent) are shown in Figure 4c–f. With increasing current, the length of the lateral secondary dendrite arms decreased, and the directionality of the dendrites also decreased. At a 700% overcurrent, the grains were almost cellular. This process can be interpreted as arc and Joule heat crystallizing and solidifying the copper wires. The grain growth of pure metals is mainly related to the heat transfer, and the heat diffusivity is much faster than the mass diffusivity [27]. At the solid–liquid interface frontier, when the temperature of the liquid phase line is higher than the actual temperature in the liquid phase and the temperature gradient is negative, a “component supercooling” zone will be formed in the liquid phase of the solid-liquid interface frontier, the solid–liquid interface is unstable, and both protuberances and pits appear at the solid–liquid interface simultaneously [28].

The “component supercooling” discriminant is shown in Equation (1):

(1) $$\frac{G_{L}}{v}=\frac{m_{L}C_{0}(1-k_{0})}{k_{0}D_{L}},$$

where *m_L_* is the liquidus slope, *C*_0_ is the solute concentration, *k*_0_ is the solute partition coefficient, *D_L_* is the liquid diffusion coefficient, *G_L_* is the liquid temperature gradient, and v is the solidification rate. It has been observed that *G_L_* and *v* affect the component undercooling and determine the grain microstructure. It is pointed out in the literature that with the decrease in $G_{L}/\sqrt{v}$, the crystal morphology changes from planar crystal to cellular crystal and equiaxial crystal [29].

At a 400% overcurrent, the metallographic image shows dendrites, and the current and arc temperature meet the following relation [30], as shown in Equation (2):

(2) $$T_{\mathrm{arc}}=4010+1658\mathrm{In}I_{a}\;\mathrm{if}\;I_{a}>\;4.5A,$$

where *T*_arc_ is the arc temperature. Equation (2) shows that the theoretical arc temperature is low with a 400% overcurrent. Therefore, the *G_L_* of the OABs is lower at 400% overcurrent, and dendritic crystals can easily be formed. Similarly, at a 700% overcurrent, the *G_L_* is larger and $G_{L}/\sqrt{v}$ is larger, and thus the cellular crystal growth is more utilized.

From Figure 5, Figure 6 and Figure 7, as the current increases, the average diameter, average perimeter, and average area gradually increase, the visible current increases and the grain shape changes. The smallest rate of change in the crystal characteristic parameters between 600–700% current indicates that crystal growth will stabilize.

### 4.3. Formatting of Mathematical Components

Figure 8 shows the 2000× microscopic morphology of the hole surface of OABs with different overcurrent intensities. In the 400% overcurrent, the inside hole is rough, the primary dendrite is keenness, and the secondary dendrite is developed. In 500% overcurrent, the secondary dendrites are less, and the tip radius of curvature decreases. When 700% overcurrent is reached, the interior holes turn smooth. This is because the OABs may be formed as a result of melting, dissolving, solidifying and other processes. The pool dissolved a large amount of the gases that were present (O_2_, H_2_O, N_2_, and CO_2_), and on the other hand, metallic oxides inside the wire also produce gases at high temperatures. When the arc was extinguished, the surface temperature of the molten pool decreased rapidly. The gases dissolved in the OABs and formed holes. However, the OABs inside remained in the liquid state due to the undercooling of the component, and the grains broke through the holes and continued to grow.

The solid–liquid interface was placed on three-dimensional coordinates, where *z* points to the liquid phase and is perpendicular to the solid–liquid interface and *x* is parallel to the solid–liquid interface. The molten OABs solid–liquid interface equation is as follows:

(3) z=δ(t)sin(ωx),

where *δ(t)* is the amplitude of the sine function and *ω* is the vibrational frequency. If the perturbation increases with time, the heat flows from the tip of the crystal to the liquid phase in large quantities [31]. This increases the velocity and pushes the tip toward the liquid phase, thus facilitating dendrite growth, and secondary dendrites grow on the primary dendrites as the perturbation intensifies. At a 400% overcurrent, the solidification time of OABs is long, and the solid-liquid interface perturbation is large; therefore, dendritic crystals easily form. At a 500% overcurrent, there were fewer secondary dendrites than at a 400% overcurrent, and the radius of curvature of the dendrite tips decreased. At a 700% overcurrent, the solidification time of OABs is short, and the solid–liquid interface perturbation is small, which hinders the growth of dendrites and eventually forms cellular crystals.

### 4.4. Effect of the Current on the EDS of OABs

According to Table 2, as the current increases, the Cu content gradually decreases and tends to be stable, while the C content gradually increases and the O and Cl elements have no obvious changes. This is because the element C is the combustion product of the PVC insulation layer, the heating rate of the conductor is slow with 400% overcurrent, and the insulation layer continues thermal decomposition under the influence of Joule heat. When an arc is generated, most of the insulation layer has been pyrolyzed and carbonized, and the combustion calorific value is low; therefore, there are less carbon substances produced by combustion. The experiment was carried out at normal temperature and pressure. After the copper wire was melted into the liquid state, O_2_ in the air would be dissolved. The O_2_ would enter the liquid metal and oxidize Cu, generating Cu_2_O and CuO, respectively. The reactive chemical formula is shown as follows:

2Cu + 1/2O_2_ = Cu_2_O, (4)

2Cu + O_2_ = 2CuO. (5)

There is no Cl element in the conductor, and thus the Cl element is the insulation thermal decomposition product. When the conductor melts it dissolves into the trace, so there are more O and Cl elements in the hole.

## 5. Conclusions

Overall, an electrical fire in a machinery plant in Xi’an city, China, was investigated. Our aim in this study was to obtain the microstructural characteristics of arc beads with various overcurrent faults and to provide a scientific reference for fire investigation. Indeed, we successfully simulated arc beads caused by overcurrent based on the fire scene. Subsequently, the effect of overcurrent faults on the grain morphology, trace elements, and microstructure of the arc beads were discussed and elucidated by OM and SEM-EDS. It was found that the metallographic characteristics of the overcurrent arc beads were dendritic crystals and cellular crystals. With the increase in current, the dendrites were gradually dissolved, while the average diameter, perimeter, and area of the grains increased. The grains were comprised of mainly cellular crystals at a 700% overcurrent. In addition, the main trace elements of the overcurrent arc beads were Cu, C, O, and Cl. When the current increased, the Cu content gradually decreased and tended to be stable. The C element gradually increased, while the O and Cl elements have no obvious changes.

## Figures and Tables

**Table 1:** Properties of the conductor and insulator used in the experiment (in an ambient atmosphere).

Characteristic.	Cu (99.99%)	Insulation (PVC)
Density (g/cm^3^)	8.9	1.4
Heat conductivity (W/m/K)	400	4
Specific heat (J/g/K)	0.39	0.5
Resistivity (Ω·m)	1.75 × 10^−8^	/
Current (I_e_) (A)	26	/
Diameter (mm^2^)	2.5	/
Melting point (K)	1357.77	160–180
Ignition temperature (K)	/	673

**Table 2:** Content of the different rated currents of copper wire.

Sample Number	Element Content (%)			
	Cu	C	O	Cl
New wire	99.95	/	0.02	/
Fire beads	87.23	9.82	2.60	0.35
400% overcurrent	85.21	11.56	2.51	0.71
500% overcurrent	81.24	16.58	1.98	0.19
600% overcurrent	80.62	17.05	2.02	0.22
700% overcurrent	80.02	18.23	1.65	0.10
